# Effect of Nucleating Agents Addition on Thermal and Mechanical Properties of Natural Fiber-Reinforced Polylactic Acid Composites

**DOI:** 10.3390/polym14204263

**Published:** 2022-10-11

**Authors:** Jae-Yeon Yang, Dong-Kyu Kim, Woong Han, Jong-Yeon Park, Kwan-Woo Kim, Byung-Joo Kim

**Affiliations:** 1 Convergence Research Division, Korea Carbon Industry Promotion Agency (KCARBON), Jeonju 54852, Korea; 2 Department of Carbon Materials and Fiber Engineering, Jeonbuk National University, Jeonju 54896, Korea; 3 Department of Chemical Engineering, Jeonbuk National University, Jeonju 54896, Korea; 4 Department of Carbon-Nanomaterials Engineering, Jeonju University, Jeonju 55069, Korea

**Keywords:** biodegradable polymer, natural fiber, nucleating agent, thermal stability, natural fiber-reinforced plastics

## Abstract

In this study, natural fiber-reinforced polylactic acid (NFRP) composite materials were prepared by adding nucleating agents (NAs) and natural fiber (NF) to compensate for the low thermal stability and brittleness of polylactic acid (PLA). The thermal stability of the fabricated composite material was investigated by differential scanning calorimetry and thermogravimetric analysis. In addition, the tensile modulus of elasticity according to the crystallinity of the composite was measured. The crystallinity of the PLA composite increased to ~700% upon the addition of the NA; thus, the thermal stability also increased. However, the changes in crystallinity and tensile modulus were insignificant when the concentration of the NA added was 4 wt.% or higher. The study demonstrates that the addition of NA and NF is effective in improving the thermal stability and mechanical properties of NFRP.

## 1. Introduction

Petroleum-based plastics, which are hard to decompose, have contributed significantly to our daily lives owing to their excellent processability, high physical properties, low specific gravity, and low price. However, a large amount of waste, including vinyl, styrofoam, and plastic containers, are disposed of in landfill or by incineration after use, causing serious environmental pollution, such as environmental hormones and air pollution [1,2,3]. Due to these problems, the automobile industry employs eco-friendly materials such as natural fibers and biodegradable polymers to fabricate the interior and exterior materials of automobile bodies. Consequently, there is a growing interest in the development and utilization of more environmentally friendly materials [4,5].

Polylactic acid (PLA) is a biodegradable polymer obtained by polymerizing natural polysaccharides such as corn and potato starch. Owing to its better processability, biocompatibility, biodegradability, and mechanical properties compared withother biodegradable polymers, it has been applied to medical products, packaging industries, and filaments for 3D printers [6,7,8,9,10,11,12,13]. Additionally, owing to the decomposition of PLA into HO_2_ and CO_2_ at the time of disposal, it is attracting considerable attention as a substitute for petroleum-based hard-to-decompose plastics [14,15,16,17]. However, the scope of its commercialization is greatly limited by the low heat resistance and low crystallinity due to slow crystallization [18,19,20,21,22,23].

The crystallinity of PLA is an important factor for mechanical and durability performances and heat deflection temperature in molding applications [24]. The addition of a nucleating agent (NA) to increase the crystallinity of PLA reduces the surface free energy barrier to nucleation and results in a higher crystallization temperature [25,26]. Harris et al. [24] demonstrated that the half-life of isothermal crystallization at 115 °C with 2 wt.% talc in PLA could be reduced by approximately 65 times compared with that of pure PLA. Nagarajan et al. [27] increased the crystallinity of PLA from 10% to 45% of that of pure PLA by adding 0.25 wt.% of LAK-301, an aromatic sulphonate derivative (potassium salt of 5-dimethyl sulfoisophthalate); crystallinity of up to 50% was achieved for LAK-301 1 wt.%.

Furthermore, another method of increasing the crystallinity of PLA is the addition of natural fibers (NF) to the polymer matrix [28,29]. The NFs can be used as a reinforcing agent in the PLA to increase the crystallinity and heat resistance and has the effect of reducing production cost by replacing a certain percentage of the matrix material with cheap NFs [30].

In this study, NA (LAK-301), an aromatic sulphonate derivative, was used in lyocell and PLA to investigate the thermal properties and crystallinity of NF-reinforced composites as a function of the added NA content. The crystallinity and thermal stability of the composite according to the LAK-301 content were studied, and the effect of the increased composite crystallinity on the mechanical properties was determined.

## 2. Experiment

### 2.1. Materials

PLA (PLA-2003D, Nature works, Minneapolis, MN, USA), NA (dimethyl 5-sulfoisophthalic acid potassium salt, LAK-301, Takemoto oil & fat Co. LTD., Gamagori-shi, Japan), and NF (Lyocell, Hyosung, Seoul, Korea) were used in the experiments.

### 2.2. Specimen Preparation

All the composites were prepared by blending in a lab-scale homemade internal mixer. To prepare the composite, PLA, NA, and NF were mixed in a tailored ratio and melt-blended at 180 °C in a mixing chamber temperature with a screw speed of 250 rpm for 30 min in a lab-scale home-built internal mixer. The total weight ratio of matrix to fillers was fixed at 4:1, and the NA was mixed in the PLA polymer in ratios ranging from 2 to 6 phr. Table 1 presents the various mixing ratios of the PLA/NA/NF in the composites. After melt-blending, each sample was molded by hot press using the vacuum bag molding method. The optimized processing temperature, time, and pressure were 180 °C, 15 min, and 10 MPa, respectively.

### 2.3. Measurement Methods

The miscibility and calorimetry of each resin were measured by differential scanning calorimetry (DSC 204 F1 Phoenix, Netzsch, Selb, Germany). Approximately 7 mg of each sample was used for the analysis. All the samples were heated at a constant rate of 10 °C/min up to 200 °C and then cooled under the same conditions (−10 °C/min up to room temperature). The chamber in which the sample was kept was purged with nitrogen gas at a flow rate of 20 mL/min to maintain an inert environment.

The thermal stability of the fabricated composites was determined in a nitrogen atmosphere at a heating rate of 10 °C/min up to 800 °C using a thermogravimetric analyzer (TGA 550, TA Instruments, New castle, PA, USA).

The different structures of the composites treated under different NA contents were determined using a wide-angle X-ray diffractometer (XRD, EMPYREAN, PANalytical LTD.,Worcester, UK), employing an EMPYREAN X-ray diffractor with a customized auto-mount and a CuKα radiation source at 40 kV and 30 mA. Diffraction patterns were recorded for the diffraction angles ranging from 10° to 60° at a speed of 2 °/min.

The tensile modulus of NF-reinforced PLA composites was measured using a universal test machine (5982, Instron LTD., Norwood, MA, USA), and the test method for studying the tensile properties of composites was conducted in accordance with ASTM D638-14 type I. The testing grip pressure and loading speed were set to 20 bar and 5 mm/min, respectively. The average values were calculated after measuring the tensile properties of five specimens under each condition.

The fractured surfaces of the tensile modulus test samples were examined by scanning electron microscopy (SEM, CX-200TA, Coxem, Deajeon, Korea) with an excitation voltage of 20 kV. The fractured surfaces were cleaned with alcohol to eliminate any impurities before the test and coated with a thin platinum layer by evaporation to improve conductivity.

## 3. Results and Discussion

### 3.1. Thermal Properties and Crystallinity after the Addition of NA

PLA has poor thermal stability and impact resistance due to its low crystallinity and slow crystallization rate, making it difficult to use in fields requiring good thermal and mechanical properties. To improve the thermal stability and mechanical properties of PLA, it is necessary to add a NA that can accelerate the crystallization as well as endow high crystallinity [31,32].

**Figure 1:**
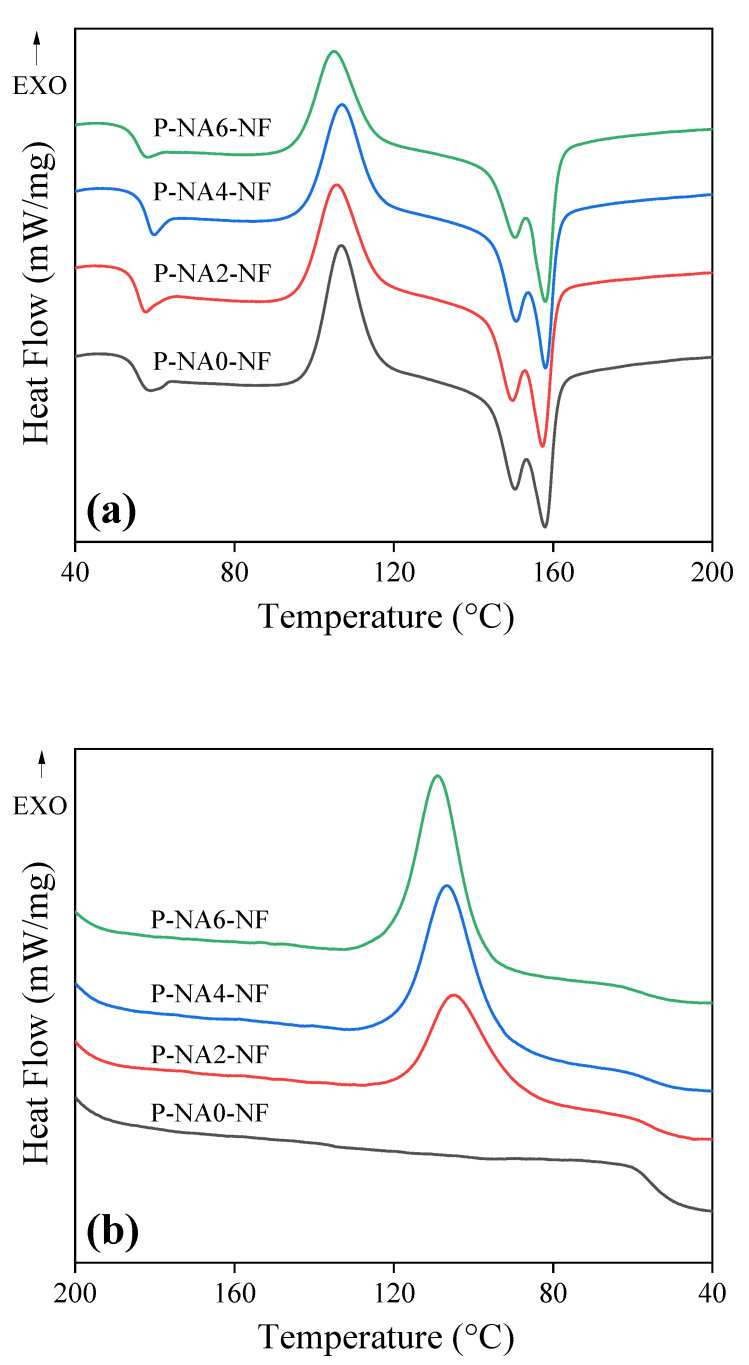

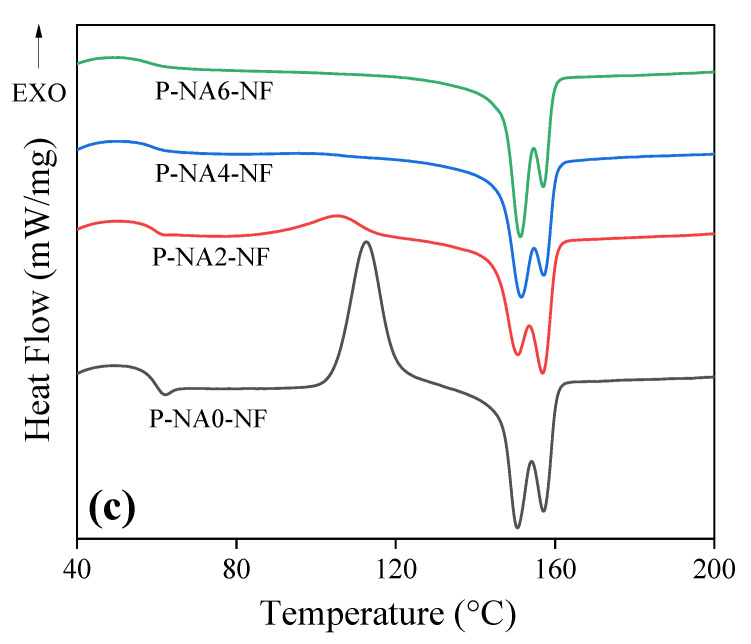
DSC thermograms of the NF-reinforced PLA composites with different NA contents; DSC curves of (**a**) first heating cycle, (**b**) cooling cycle, and (**c**) second heating cycle.

PLA crystallizes in approximately 2 h in the temperature range of 90–120 °C, between its T_g_ (glass transition temperature) and T_m_ (melting temperature). NA was added to compensate for these shortcomings, and the DSC curves of the PLA composites as a function of the added NA content are shown in Figure 1.

As shown in Figure 1b, crystallization peaks were observed during cooling for the PLA composites containing NA. In the case of the PLA composite that did not contain NA, no peak corresponding to T_c_ (crystallization temperature) could be observed during secondary heating. In the case of P-NA2-NF, the structures that were not crystallized during the cooling process showed a fine crystallization peak during secondary heating. During the cooling process, the extent and rate of crystal formation increased with increasing NA content [22,31,32]. In addition, as shown in Figure 1c, double peaks corresponding to melting were observed in the DSC curves of the PLA composites containing the NA. The crystallinity improved upon the addition of a NA, and it can be inferred that crystals with a shape different from that of the conventional PLA available were generated.

**Figure 2:**
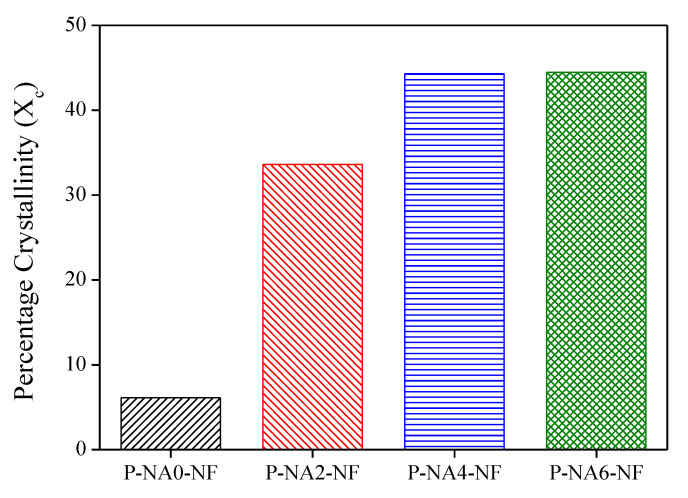
Percentage crystallinity of the NF-reinforced PLA composites with different NA contents.

An equation was used to obtain the percentage crystallinity (*X_c_*) of composites, and the relationship between the NA contents and crystallinity is shown in Figure 2. The method uses Equation (1)

(1) $$X_{c}=\left[\frac{\Delta H_{m}-\Delta H_{cc}}{\emptyset \times f.\Delta H_{m}^{0}}\right]\times 100\%$$

Here, Δ*H*_*m*_ represents the melting enthalpy; Δ*H*_*cc*_ is the cold crystallization enthalpy; *f*.Δ$H_{m}^{0}$ is the 100% crystalline PLA’s melting enthalpy (93.7 J/g). ∅ is the weight fraction of PLA in the composites [22,32].

Figure 2 and Table 2 shows the crystallinity calculated according to Equation (1) based on the DSC data. For PLA composites with added NA, the crystallinity increased more than that of P-NA0-NF. The size of the PLA crystal becomes smaller as the NA is added; however, the crystallinity did not increase for P-NA6-NF even when 6 phr of the NA was added [33]. Thus, there is a critical minimum concentration of the NA. Above this concentration there is no significant change in the crystallization rate due to the motion of the NA. In this study, the economical amount of the NA to be added for PLA crystallization was determined to be 2 phr.

**Figure 3:**
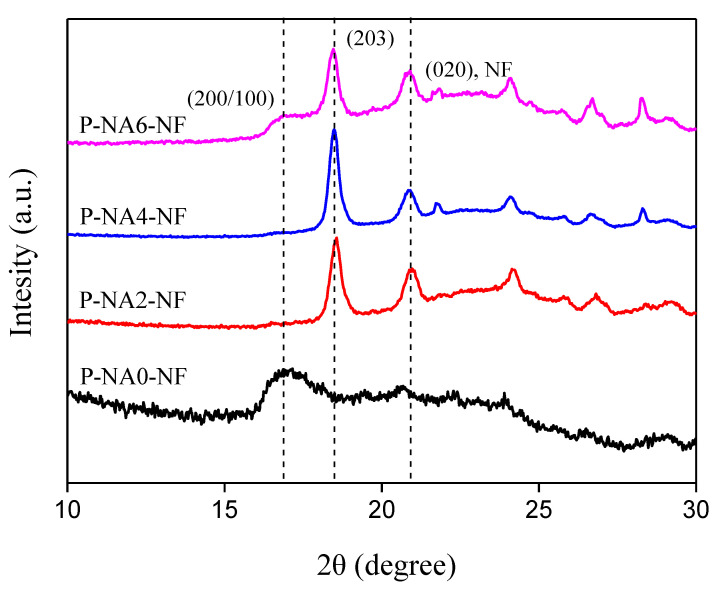
XRD patterns of the NF-reinforced PLA composites with different NA contents.

In addition to the crystallinity measurement using DSC, the reliability of crystallization was confirmed using XRD. Figure 3 shows the XRD results of the PLA composites with added NA (0, 2, 4, 6%) and without NA (P-NA0-NF). For P-NA0-NF, which did not contain NA, no peak corresponding to crystallinity was observed. For PLA composites with added NA, the percentage crystallinity (X_c_) increased, and a new crystal peak (203) appeared in the XRD pattern [4,6]. This peak appears due to the change in the crystal phase with increasing crystallization rate owing to the addition of the NA; the crystallization proceeds from the preceding to the plate shape [4,6,34].

**Figure 4:**
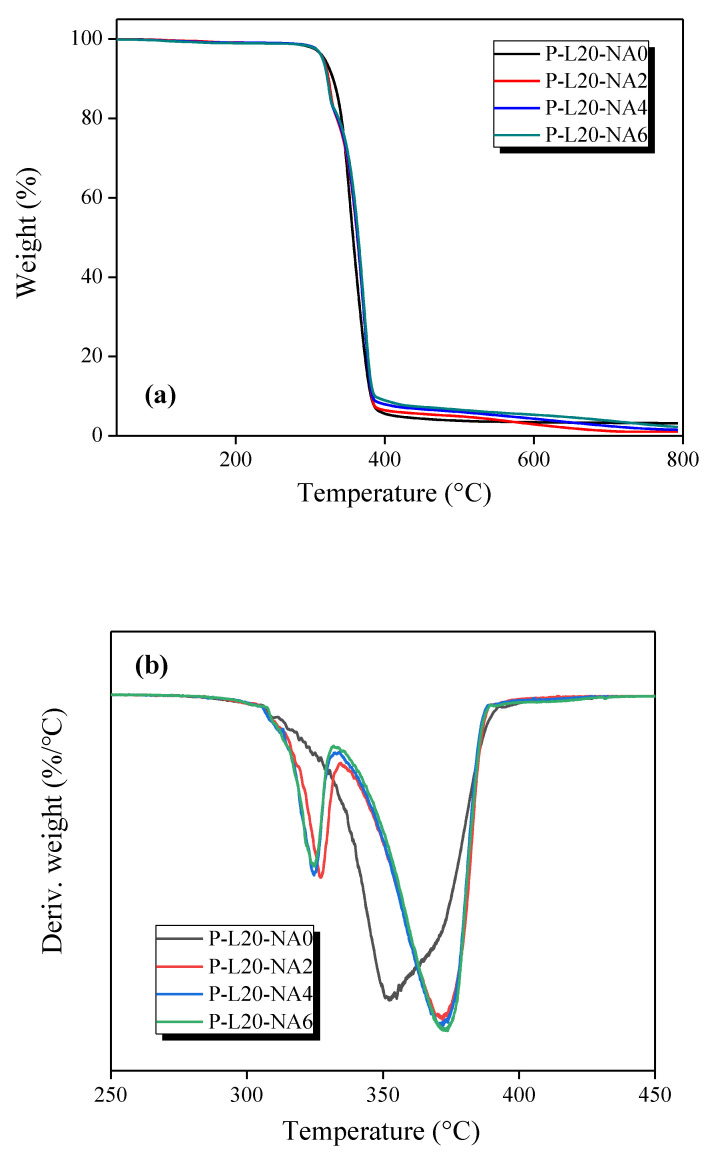
TGA (**a**) and DTG (**b**) thermograms of the NF-reinforced PLA composites with different NA contents.

Thermogravimetric analysis (TGA) is a commonly used technique for the rapid evaluation of the thermal stability of different materials, and it indicates the decomposition of polymers at various temperatures. Figure 4a shows the TGA thermograms of the NF-reinforced PLA composites with different NA contents from room temperature (28 °C) to 800 °C in a nitrogen atmosphere. Figure 4b shows the derivative TG (DTG) curves of the TGA curves, confirming the thermal decomposition of the NFRP over a certain temperature range at various NA contents. In the case of P-L20-NA0, which did not contain NA, thermal decomposition was initiated at 300 °C, and rapid decomposition occurred at 350 °C. In the case of the NFRP composite materials containing NA, thermal stability improved upon crystallization. NFRP containing NA underwent first pyrolysis at 320 °C, and the thermal stability improved compared with that of P-L20-NA0. The second pyrolysis peak was observed at 370 °C. In addition, the primary pyrolysis peak at 320 °C was considered to correspond to the decomposition of the unstable crystals of PLA.

As an NA is added, the unstable polymer in which radicals are formed is initially thermally decomposed. However, more stable pyrolysis is observed at higher temperatures, and the final yield also increases.

In an in-depth analysis, the integral procedure decomposition temperature (IPDT) proposed by Doyle [35] has been correlated with the volatile parts of polymeric materials and used to estimate the inherent thermal stability of polymeric materials [36]. The IPDT was calculated as follows:

(2) $$\mathrm{IPDT}\left({}^{\circ}C\right)=A^{*}\cdot K^{*}\left(T_{f}-T_{i}\right)+T_{i}$$

(3) $$A^{*}=\frac{S_{1}+S_{2}}{S_{1}+S_{2}+S_{3}}$$

(4) $$K^{*}=\frac{S_{1}+S_{2}}{S_{1}}$$

Here, A* is the area ratio of the total experimental curve and the total TGA thermogram; *T_i_* is the initial experimental temperature. *T_f_* is the final experimental temperature. Figure S1 shows a representation of S_1_, S_2_, and S_3_ for calculating A* and K*. The thermal stability results calculated according to the above method are presented in Table 3, where IDT is the initial polymer decomposition temperature, and T_max_ is the maximum pyrolysis temperature.

The IPDT of the P-L20-NA0 sample was 374.20 °C, and the IPDTs of the composites with added NA were higher than that of pure composite. The thermal stability of NF-based PLA composites increased with the increasing content of NA. These results indicate that NA can increase the thermal stability of NF-based PLA composites, which is due to the increase in PLA crystallinity.

### 3.2. Modulus of Elasticity after the Addition of NA

**Figure 5:**
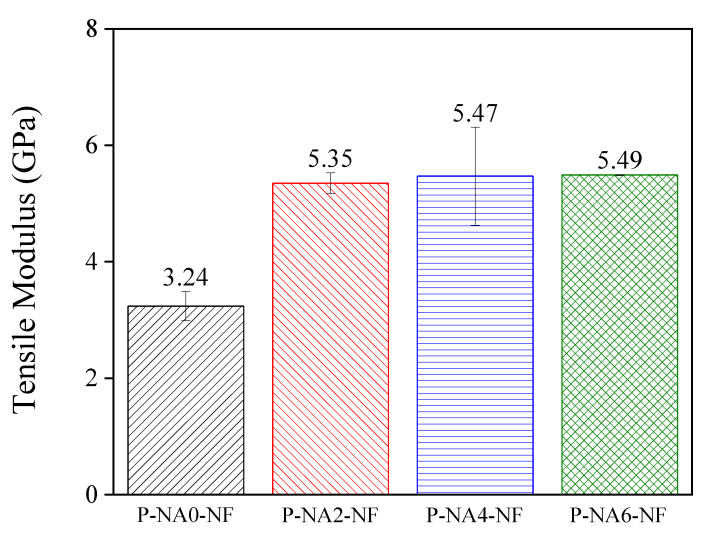
Tensile modulus of the NF-reinforced PLA composites with different NA contents.

Figure 5 shows the mechanical properties of PLA composites according to the amount of NA. The NAs were added to improve the physical properties of the PLA composites. By optimizing the ratio, the effect of the NA on the tensile modulus was determined. The P-NA2-NF sample with 2 phr of added NA showed a higher tensile modulus than P-NA0-NF, and as the NA content increased, it had no significant effect on the tensile modulus [37,38].

**Figure 6:**
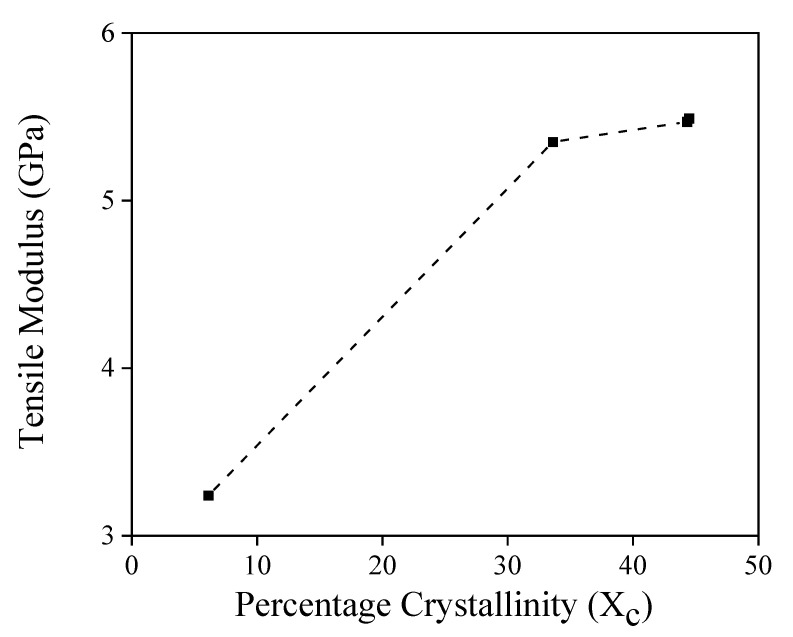
Tensile modulus of the NF-reinforced PLA composites with different NA contents as a function of percentage crystallinity.

Additionally, as shown in Figure 6, when different amounts of NAs were added, the crystallinity and crystallization rate increased, and the mechanical properties changed. Consequently, in a crystalline resin such as PLA, the degree of crystallinity increases in the process of forming a solid from the molten state through cooling. The tensile modulus is improved by adding the NA, and the crystallinity is improved by refining the crystal size. As the crystallization temperature of PLA increases, the overall crystallization rate increases, making it possible to reduce the cooling time when molding the PLA composites. NAs have a critical minimum concentration, depending on the type of NA, above which no significant effect on the crystallization rate is observed due to the addition of NA [33,36,37,38].

**Figure 7:**
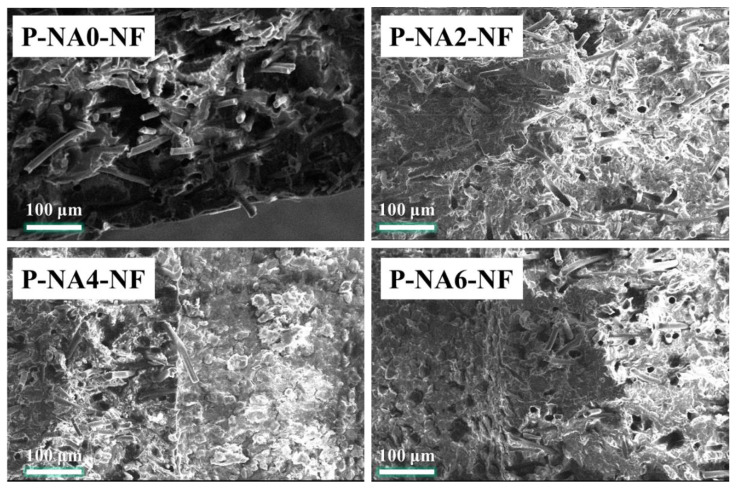
Scanning electron microscopy images of the fractured surfaces of NF-reinforced PLA composites with different NA contents.

After the tensile modulus test, the fractured section of each sample was observed by scanning electron microscopy, and the results are shown in Figure 7. 

The morphology of the fractured surfaces of the specimens differs from each other. The fractured surface of P-NA0-NF differs from that of samples with NA added because of the higher tensile modulus of PLA composites in the NA2, NA4, and NA6 systems. The sample with added NA is clearly broken, whereas the sample with no added NA breaks in lumps.

## 4. Conclusions

Through thermal analysis, this study demonstrates that the crystallinity of PLA, a biodegradable polymer, can be improved with the addition of an NA (LAK-301). Although pure PLA is biodegradable and eco-friendly, its crystallinity, as well as the rate of crystallization, are low. The proportion of the cooling process increases during the actual composite material molding or injection molding, because of which the overall cycle time increases. Therefore, we added NA, which was expected to improve the crystallinity. The overall shorter cycle time and an improved degree of crystallinity, as demonstrated in this study, render this material a strong competitor against general-purpose resins. The effect of NA content on the thermal and mechanical properties of the NF-reinforced PLA composites was investigated. NAs can create nucleation sites and promote the crystallization of PLA. The thermal analysis confirmed that crystallization was accelerated upon adding NA. In the case of P-NA4-NF containing 4 phr of NA, the X_c_ value was the highest (44.31). There was no significant effect on crystallization rate above a certain amount of NA. A critical minimum concentration was considered to exist due to the motion of the NA, and the most economical amount of NA for endowing good mechanical properties to the material was determined to be 2 phr. The degree of crystallinity is increased due to the presence of a NA during the transformation from the molten state to the solid state after cooling so superior thermal stability and mechanical properties compared with those of raw PLA can be achieved.

## Figures and Tables

**Table 1:** PLA/NA/NF formulation for the composite preparation.

Samples	PLA (wt.%)	NA (phr)	NF (wt.%)
P-NA0-NF	80	0	20
P-NA2-NF	80	2	20
P-NA4-NF	80	4	20
P-NA6-NF	80	6	20

**Table 2:** Thermal characterization of the NF-reinforced PLA composites with different NA contents.

Samples	^1^^st^T_m_ (°C)	^2^^nd^T_m_ (°C)	ΔH_m_ (J/g)	T_mc_ (°C)	^1^^st^T_cc_ (°C)	^2nd^T_cc_ (°C)	ΔH_cc_ (J/g)	X_c_ (%)
P-NA0-NF	150.4 /158.0	150.6 /157.1	34.16	-	106.9	112.7	29.58	6.11
P-NA2-NF	149.9 /157.4	150.6 /156.9	32.93	105.0	105.8	105.3	7.733	33.61
P-NA4-NF	150.6 /158.0	151.5 /157.2	34.05	106.7	107.1	-	-	44.31
P-NA6-NF	150.5 /158.1	151.3 /157.0	33.33	109.2	105.1	-	-	44.46

**Table 3:** Thermal stability parameters of the NF-reinforced PLA composites with different NA contents.

Sample Name	IDT (°C)	T_max_ (°C)	A*·K*	IPDT (°C)
P-NA0-NF	414.48	351	0.581	374.20
P- NA2-NF	494.88	369	0.603	380.86
P- NA4-NF	561.54	370	0.622	386.69
P- NA6-NF	626.92	372	0.635	390.45

## Data Availability

Not applicable.

## Supplementary Materials

The following supporting information can be downloaded at: https://www.mdpi.com/article/10.3390/polym14204263/s1, Figure S1: Schematic of S_1_, S_2_, and S_3_ for *A** and *K**.
