# Supplementary figures and images for: Effect of Nucleating Agents Addition on Thermal and Mechanical Properties of Natural Fiber-Reinforced Polylactic Acid Composites
Source: Polymers (Basel). 2022 Oct 11;14(20):4263. doi: 10.3390/polym14204263 (PMC9607137; doi:10.3390/polym14204263)

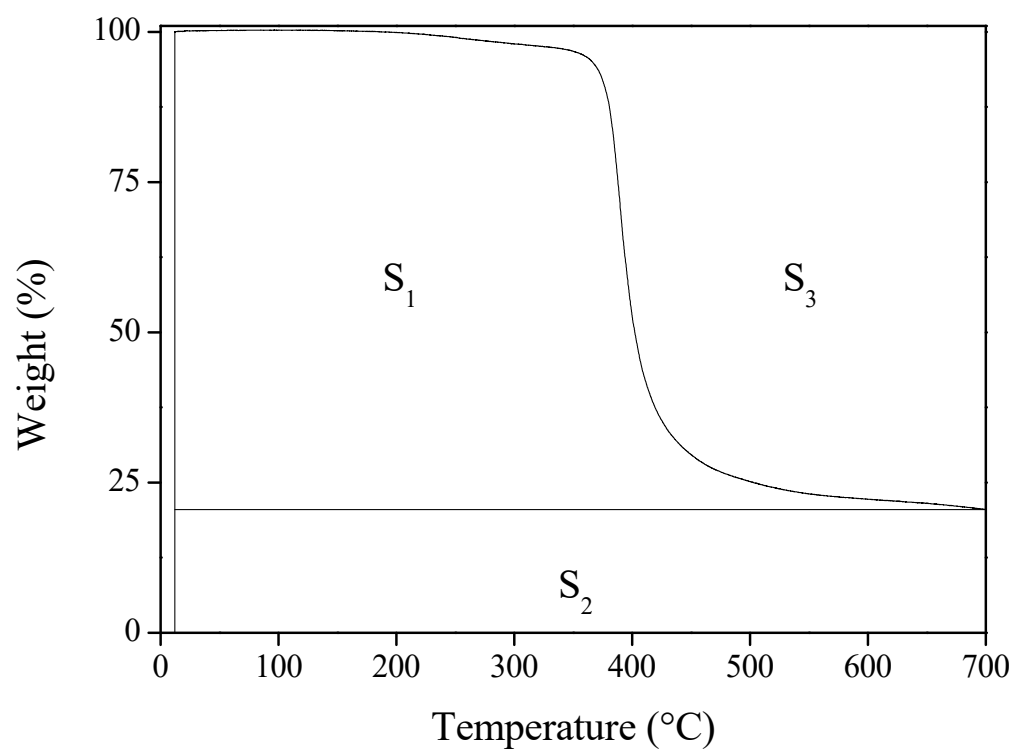

**Figure S1.** Schematic of  $S_1$ ,  $S_2$ , and  $S_3$  for  $A^*$  and  $K^*$ .

Supplement: Supplementary file 1 [file polymers-14-04263-s001.zip › polymers-1918558-supplementary.pdf]
